# One-Pot Multicomponent Polymerization, Metal-, and Non-Metal-Catalyzed Synthesis of Organoselenium Compounds

**DOI:** 10.3390/polym14112208

**Published:** 2022-05-29

**Authors:** Saad Shaaban, Hany M. Abd El-Lateef, Mai M. Khalaf, Mohamed Gouda, Ibrahim Youssef

**Affiliations:** 1 Department of Chemistry, College of Science, King Faisal University, P.O. Box 380, Al-Ahsa 31982, Saudi Arabia; hmahmed@kfu.edu.sa (H.M.A.E.-L.); mmkali@kfu.edu.sa (M.M.K.); mgoudaam@kfu.edu.sa (M.G.); 2 Department of Chemistry, Organic Chemistry Division, College of Science, Mansoura University, Mansoura 11432, Egypt; 3 Chemistry Department, Faculty of Science, Sohag University, Sohag 82524, Egypt; 4 Transcranial Focused Ultrasound Laboratory, UTSW Medical Center, Dallas, TX 75390, USA; 5 Neuroradiology and Neuro-Intervention Section, Department of Radiology, UTSW Medical Center, Dallas, TX 75390, USA

**Keywords:** polymerization, organoselenium, one-pot multicomponent, metal-free-catalyzed

## Abstract

The one-pot multicomponent synthetic strategy of organoselenium compounds represents an alternative and robust protocol to the conventional multistep methods. During the last decade, a potential advance has been made in this domain. This review discusses the latest advances in the polymerization, metal, and metal-free one-pot multicomponent synthesis of organoselenium compounds.

## 1. Introduction

**Figure 1:**
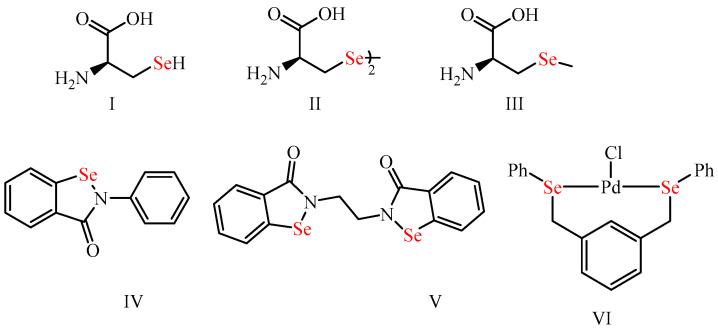
Organoselenium compounds (**I**–**VI**) with potential biological activities and diverse applications.

Organoselenium (OSe) compounds have recently gained considerable interest as a potential class of organic motifs due to their outstanding applications in synthetic organic and medicinal chemistry and their possible properties in materials science [1,2,3,4]. These are attributed to the exceptional properties of the selenium (Se) element. The latter is a non-metal present in almost living organisms as part of selenoproteins (e.g., thioredoxin reductases and glutathione peroxidase antioxidants enzymes) [5,6,7,8,9]. Accordingly, Se is crucial for protecting human cells from oxidative damage and the immune system’s normal function [10,11,12]. Compared to sulfur (S), Se has a larger atomic radius (S: 1.02 Å vs. Se: 1.17 Å), lower electronegativity (S: 2.58 vs. Se: 2.55), higher polarizability (S: 2.9 Å vs. Se: 3.8 Å), and therefore Se is a likely better nucleophile than the S [5,8,13]. Accordingly, OSe compounds are known for their ability to react with O_2_-free radicals and thus prevent the progression of oxidative stress-related diseases [3,6,10,14]. Furthermore, OSe compounds were also used in material science due to their semiconductor potential and therefore used in sodium-ion batteries, solar cells, and H_2_ evolution catalysts [2,15,16,17]. Moreover, the Se center is present in many naturally occurring and bioactive interesting OSe compounds (Figure 1), such as the selenoaminoacids (e.g., selenocysteine (**I**), selenomethionine (**II**), and selenocystine (**III**)) [18,19,20]. Furthermore, ebselen (**IV**) is the most investigated Se compound with exciting GPX-like activity and has recently reached clinical phase III trials as a possible drug for Meniere’s disease [11,21,22] (Figure 1). Moreover, ethaselen (**V**) entered trial phase II for non-small lung cancer treatment [23,24,25,26]. On the other hand, different OSe compounds have also manifested efficient catalytic activity for various organic reactions, such as the palladium-based OSe complex **VI** for the Heck reaction (Figure 1) [27].

Given the exciting activities and the diverse applications of the OSe compounds, sustainable and efficient approaches for their preparation are in high demand. The synthesis of OSe compounds depends on their chemical structures (e.g., selenides, selenocyanates, diselenides) [26,28,29]. Standard methods include direct selenylation via reaction with proper Se reagents such as Na_2_Se_2_ and KSeCN. On the other hand, indirect selenylations include rearrangement of Se-containing precursors (e.g., isoselenocyanates) or multistep synthetic procedures using elemental Se together with organolithium or Grignard reagents and [2,13,24,30,31,32,33,34,35]. Despite being efficient, these classical strategies are relatively limited due to the challenges associated with the complicated synthetic procedure, regioselectivity, or harsh reaction conditions issues. Recently, various alternative reactions were developed as efficient and milder synthetic protocols within combinatorial chemistry (CC) [36,37,38,39,40,41,42,43]. The latter has emerged as a robust tool in medicinal chemistry [44,45]. It is now widely and consecutive covalent bonds formed between different building blocks [46]. Concurrently, drug candidates are discovered and selected by the screening of small molecule libraries for particular biological targets [47]. Despite the various strategies used in CC, multicomponent reactions (MCRs) are amongst the most investigated techniques for the efficient synthesis of chemical libraries [43,45]. MCRs have been known for over a century. They include generating skeletally diverse and complex molecular entities from more than two starting materials by straightforward chemical transformations employing comparatively mild conditions [37,48,49,50].

From an economical step and atom viewpoint, the MCR one-pot strategy would offer more robust access to OSe compounds. Though the MCRs have emerged as an efficient tool for constructing C-S bonds, similar approaches for synthesizing C-Se bonds have recently attracted more attention. We here want to summarize the recent developments of OSe compounds using the MCR one-pot approach to pave the way for medicinal chemists to have more accessible synthetic access to such a biologically relevant category of compounds. Specifically, we have structured this review based on the developments in metal-catalyzed and metal-free reactions in addition to the multicomponent polymerization synthesis of OSe compounds.

## 2. The One-Pot Multicomponent Combinatorial Synthesis of OSe Compounds

### 2.1. Metal-Catalyzed Synthesis of the OSe Compounds

**Scheme 1:**
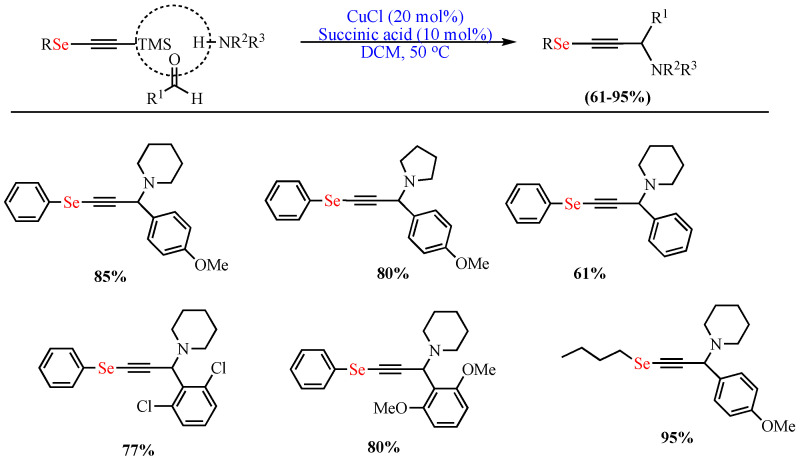
Synthesis of OSe propargylamines.

In 2013, de Oliveira et al. reported the one-pot Cu-catalyzed (CuCl) synthesis of OSe propargylamines in excellent yields (up to 95%) via A3-coupling of trimethylsilyl Se-acetylene, *p*-methoxybenzaldehyde, and piperidine catalyzed in DCM as the solvent and in the presence of succinic acid additive at 50 °C (Scheme 1) [51].

**Scheme 2:**
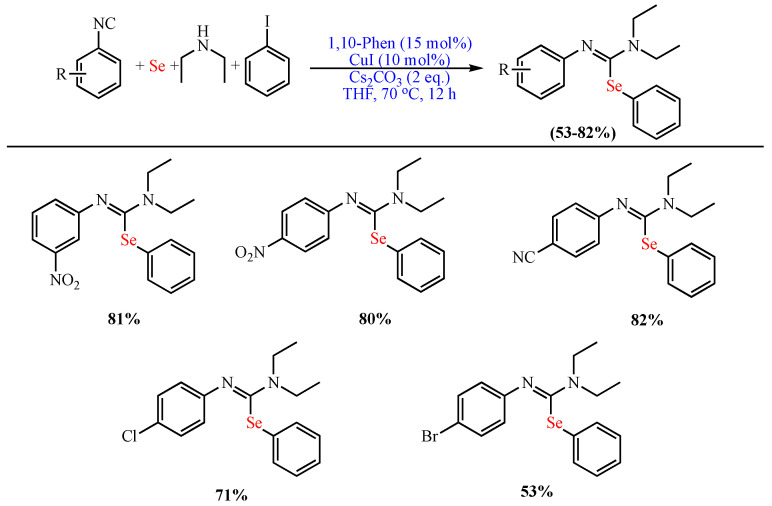
Synthesis of functionalized isoselenoureas.

In 2017, Liu et al. reported the synthesis of highly functionalized isoselenoureas through the Cu-catalyzed (CuI) 1,10-phenanthroline-promoted MCR of elemental Se, aryl iodides, isocyanides, and amines using Cs_2_CO_3_ as the base and THF as the solvent at 70 °C (Scheme 2) [52].

**Scheme 3:**
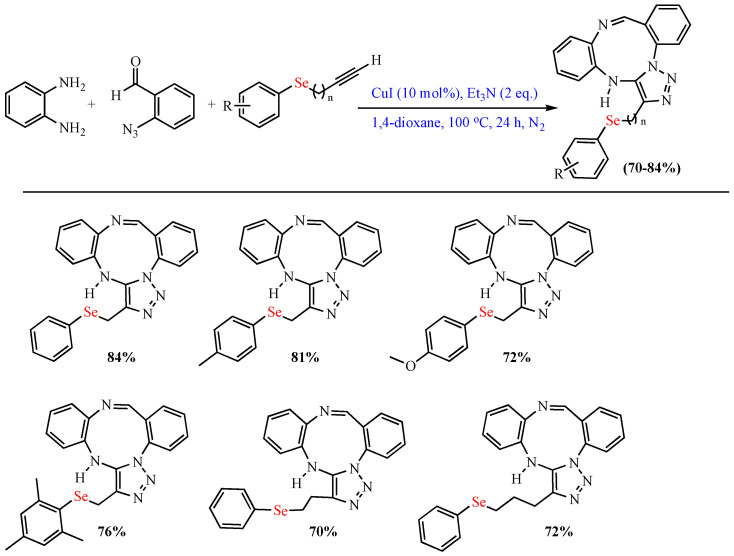
Synthesis of (arylselanyl)-alkyl-1,2,3-triazolo-1,3,6-triazonines.

In 2018, Aquino et al. synthesized (arylselanyl)-alkyl-1,2,3-triazolo-1,3,6-triazonines via CuI-catalyzed MCR of 2-azidobenzaldehyde, 1,2-diaminobenzene, and various arylchalcogenyl alkynes in dioxane at 100 °C. The reaction included a wide variety of arylchalcogenyl alkynes (Scheme 3) [53].

**Scheme 4:**
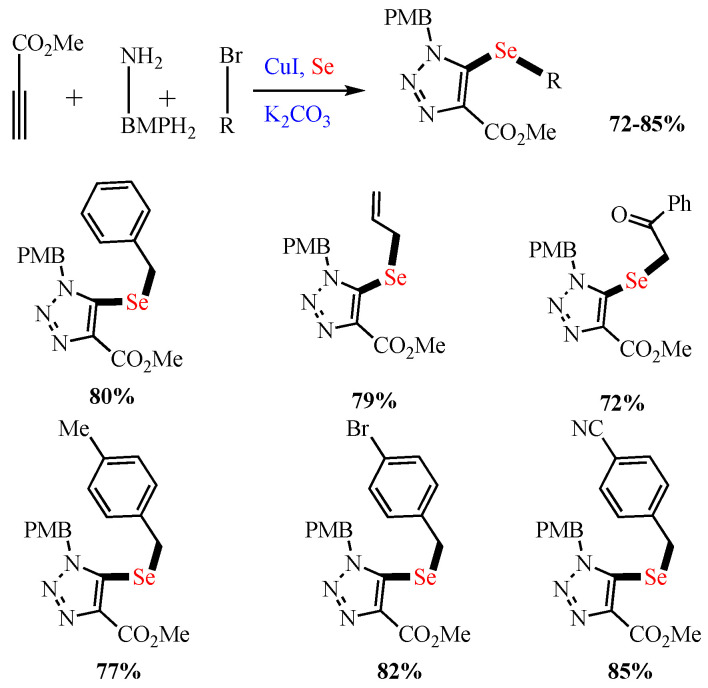
Synthesis of 5-selenotriazoles.

Zhang et al. reported the sequential multistep Cu-catalyzed (CuI) assembly of 5-selenotriazoles via the one-pot reaction of elemental Se, azide, alkyl halide, and alkyne. The selenylating agent and Cu(I) triazolides were generated in situ*,* and the reaction proceeded under mild conditions using readily available substrates with broad variety scope in high regioselectivity (Scheme 4) [54].

**Scheme 5:**
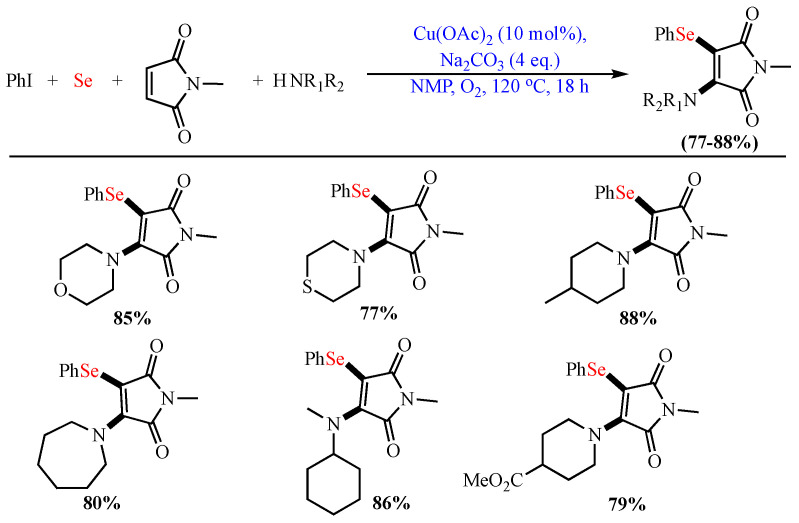
Aminoarylselenation of maleimides.

In 2019, Gao et al. reported the Cu-catalyzed (Cu(OAc)_2_) cross-coupling oxidative aminoarylselenation of maleimides using elemental Se, aryl iodides, and secondary amines. This reaction enabled the bifunctionalization of alkenes via the simultaneous one-pot construction of the C−N bond and C−Se bonds (Scheme 5) [55].

**Scheme 6:**
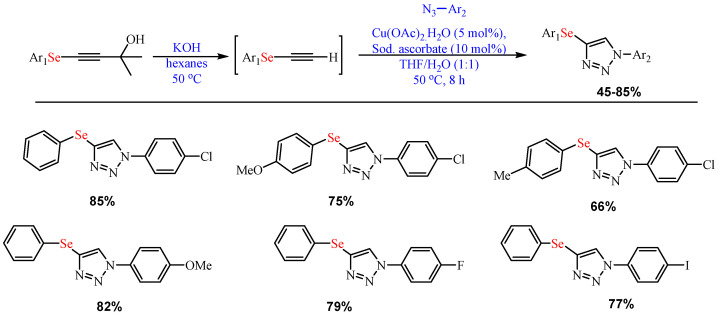
Synthesis of 4-arylselanyl-1*H*-1,2,3-triazoles.

Recently, Begini et al. developed 4-arylselanyl-1*H*-1,2,3-triazoles from the one-pot Cu-catalyzed (Cu(OAc)_2_) reaction of selanylalkynylcarbinols and aryl azides in the presence of sodium ascorbate (10 mol%) in H_2_O and THF (1:1) mixed solvent at 50 °C (Scheme 6) [56].

**Scheme 7:**
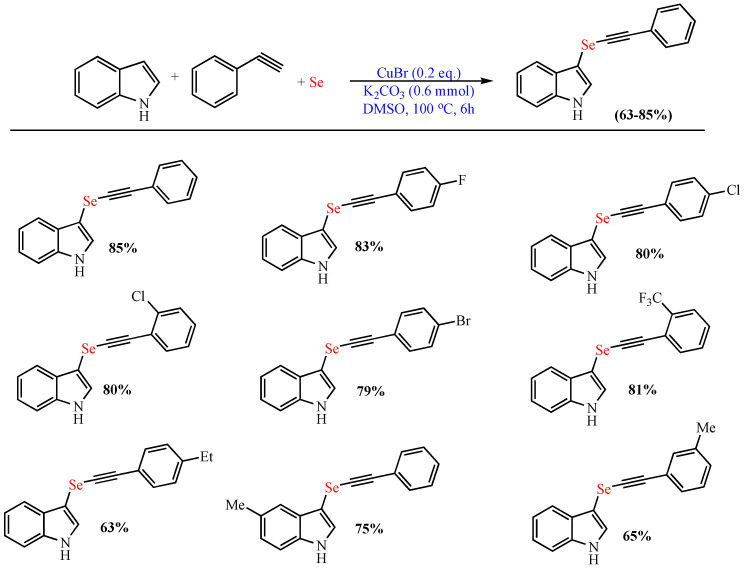
Synthesis of 3-((arylethynyl)selanyl)-1*H*-indoles.

In 2021, Rather et al. disclosed the Cu-catalyzed (CuBr) synthesis of 3-((arylethynyl)selanyl)-1*H*-indoles in good yield (up to 83%) from elemental Se phenylacetylene and indole using K_2_CO_3_ as the base and DMSO as the solvent at 100 °C. The strategy tolerates different indoles and phenylacetylene motifs and can be expanded to a gram scale without any difficulties (Scheme 7) [57].

**Scheme 8:**
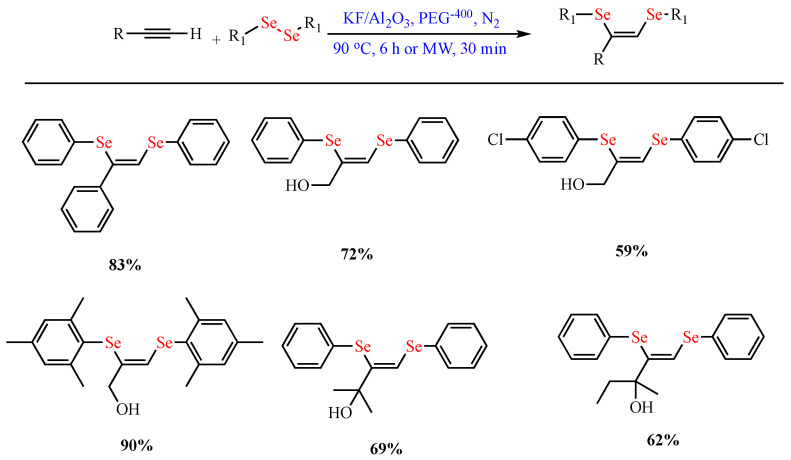
Synthesis of (*Z*)-1,2-bis-arylselanyl alkenes.

Furthermore, Lara et al. reported the synthesis of (*Z*)-1,2-bis-arylselanyl alkenes by the one-pot reaction of terminal alkynes with diaryl diselenides using KF/Al_2_O_3_ and PEG-400 as a solvent in good yields. Interestingly, the reaction time was reduced from 6 h under conventional conditions to 30 min using microwave irradiation (Scheme 8) [58].

**Scheme 9:**
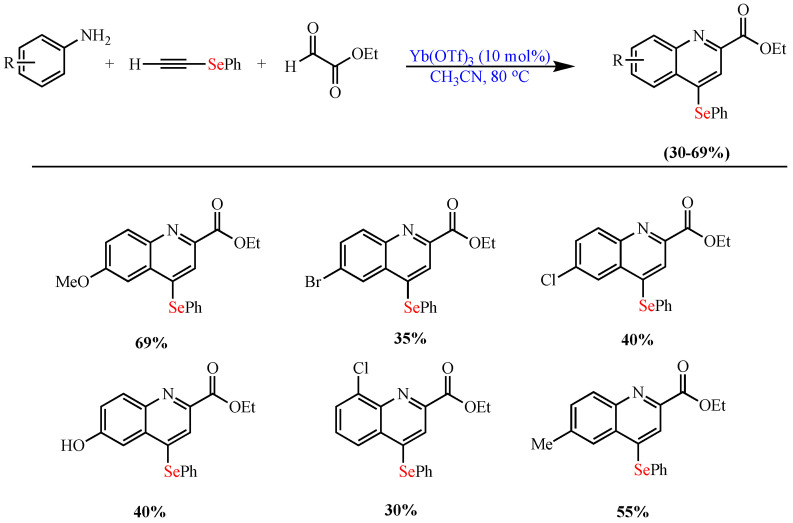
Synthesis of 2,4- disubstituted Se-quinoline derivatives.

De Oliveira et al. reported the ytterbium(III)-catalyzed (Yb(OTf)_3_) synthesis of 2,4- disubstituted Se-quinoline derivatives via Povarov MCR between ethyl glyoxylate, *p*-anisidine, and ethynyl(phenyl)selane in CH_3_CN as the solvent at 80 °C and in moderate yields (up to 69%) (Scheme 9) [59].

**Scheme 10:**
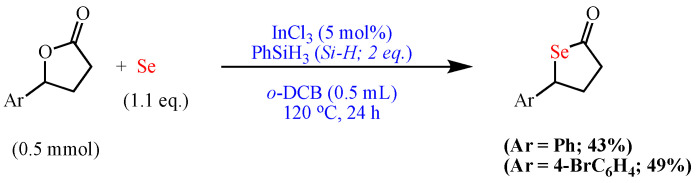
Synthesis of selenolactones.

Moreover, Sakai et al. reported the indium-catalyzed (InCl_3_/PhSiH_3_) one-pot synthesis of selenolactones from elemental Se and lactones using *o*-dichlorobenzene as the solvent at 120 °C for 24 h (Scheme 10) [60].

**Scheme 11:**
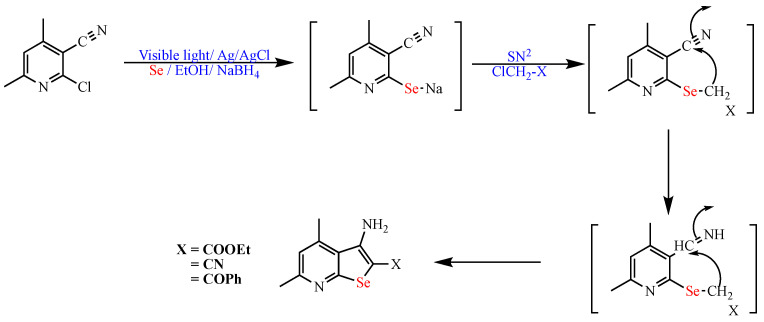
Synthesis of seleno [2,3-b]pyridine derivatives.

Recently, Attia et al. reported the one-pot synthesis of seleno [2,3-b]pyridine derivatives using Ag/AgCl nanoparticles under visible light irradiation. The reaction was carried out under mild conditions using visible light as the energy source, Ag/AgCl- nanoparticles, and EtOH as the solvent. It is worth noting that the Ag/AgCl- nanoparticles showed high catalytic activity and reusability potential up to five cycles in 94–91% isolated yields (Scheme 11) [61].

**Scheme 12:**
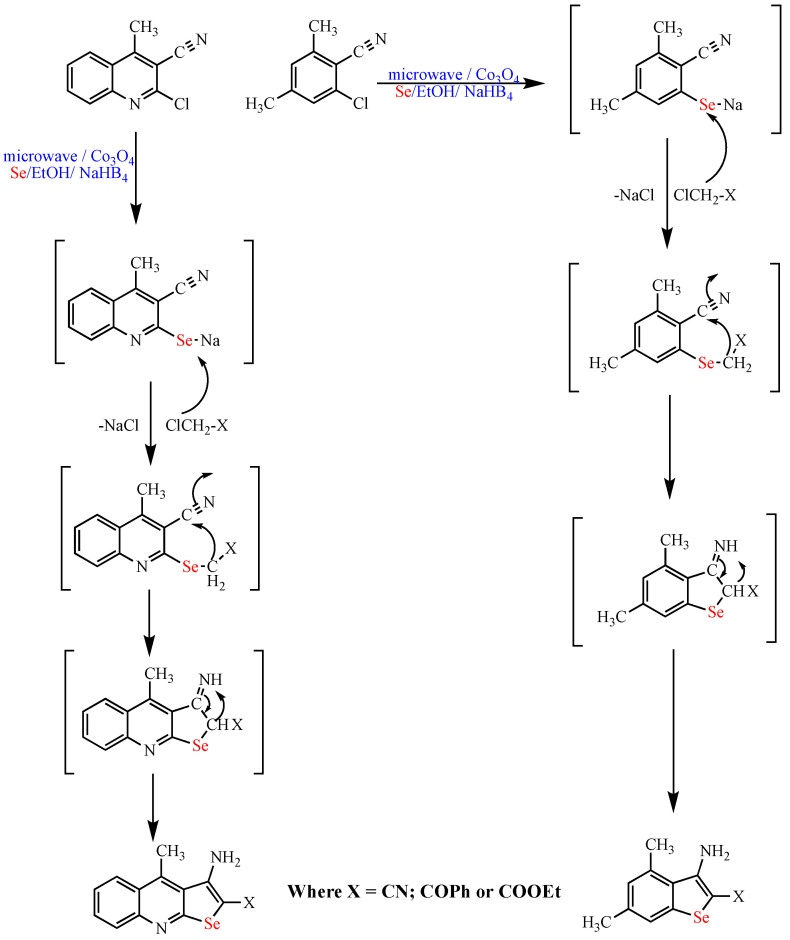
Synthesis of selenopyridine and quinoline derivatives.

Recently, the same group by Abdel-Hafez et al. reported the synthesis of selenopyridine and quinoline derivatives in excellent yields (up to 90%) and selectivity using Co_3_O_4_ nanoparticles heterogeneous catalyst under microwave irradiation and water as the solvent (Scheme 12) [62].

### 2.2. Metal-Free Synthesis of the OSe Compounds

**Scheme 13:**
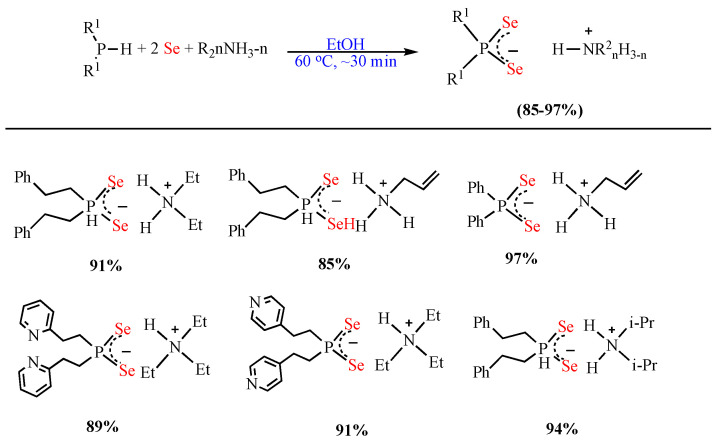
Synthesis of alkylammonium diselenophosphinates.

In 2010, Artem’ev et al. reported the MCR one-pot synthesis of alkylammonium diselenophosphinates in excellent yield (up to 97%) via reaction of elemental Se with amines (e.g., primary, secondary, or tertiary) and secondary phosphines in ethanol at 60 °C (Scheme 13) [63].

**Scheme 14:**
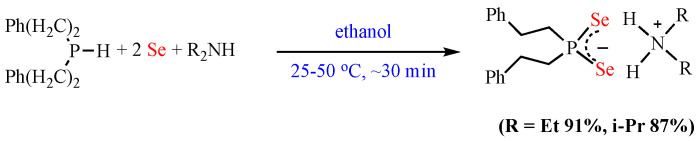
Synthesis of diselenophosphinates.

Furthermore, Artem’ev et al. also synthesized diselenophosphinates via a three-component reaction of secondary phosphine, Se powder, and amines in ethanol at 50–75 °C for 30 min (Scheme 14) [64].

**Scheme 15:**
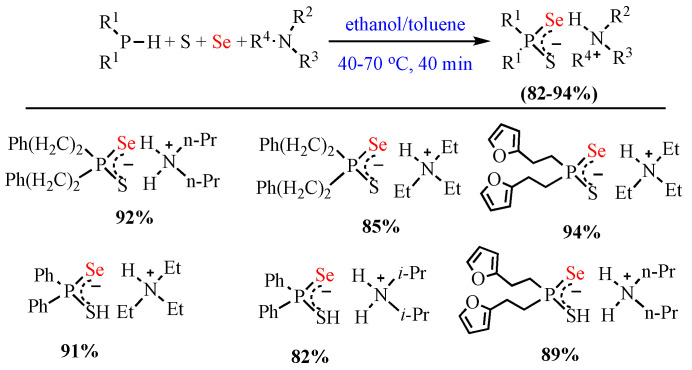
Synthesis of mono-, di-, and trialkylammonium thioselenophosphinates.

Additionally, Artem’ev et al. reported the one-pot multicomponent synthesis of mono-, di-, and trialkylammonium thioselenophosphinates in good yields (up to 94%) from secondary phosphanes, amines (primary, secondary, or tertiary), elemental S, and elemental Se (Scheme 15) [65].

**Scheme 16:**
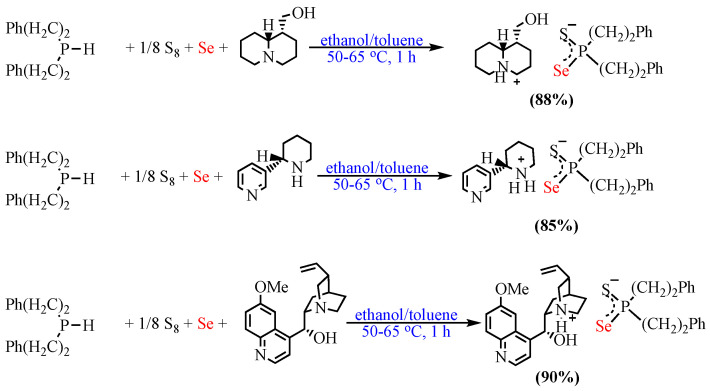
Synthesis of mono-, di-, and trialkylammonium thioselenophosphinates of lupinine, anabasine, and quinine.

Despite the high yield and considerable success of the above reaction, it was limited to certain amines such as triethyl amine, dipropyl amine, and diisopropylamine. In 2012, Artem’ev et al. extended this reaction to natural alkaloids of different *N*-bases, namely, lupinine, anabasine, and quinine [66] (Scheme 16).

**Scheme 17:**
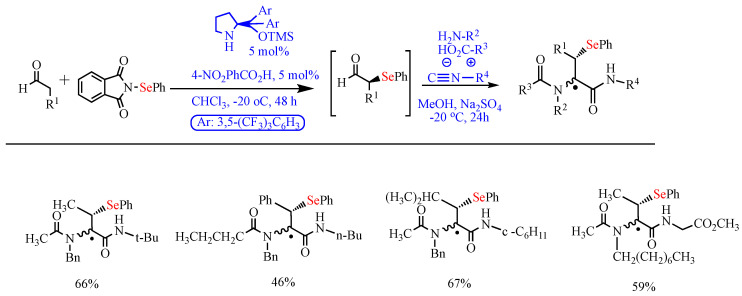
Synthesis of selenocysteine-based peptoids and peptide–peptoid conjugates.

De La Torre et al. reported the metal-free synthesis of new selenocysteine-based peptoids and peptide–peptoid conjugates. The latter includes organocatalytic insertion of phenylselenium into the backbone of the aldehyde moiety using Jørgensen’s catalyst, followed by a subsequent Ugi reaction (Scheme 17) [67].

**Scheme 18:**
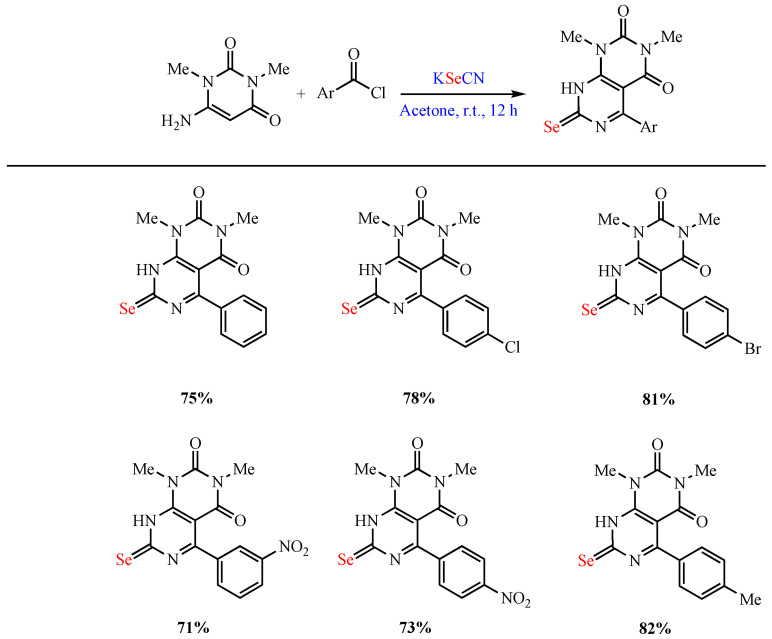
Synthesis of 5-aryl-1,3-dimethyl-7-selenoxopyrimidino [4,5-d]pyrimidine-2,4(1H,3H)-diones.

In 2018, Singh et al. reported the one-pot synthesis of 5-aryl-1,3-dimethyl-7-selenoxopyrimidino [4,5-d]pyrimidine-2,4(1H,3H)-diones in good yields (up to 82%) by the reaction of aroyl chloride, KSeCN and 6-amino-*N*,*N*′-dimethyluracil in acetone (Scheme 18) [68].

**Scheme 19:**
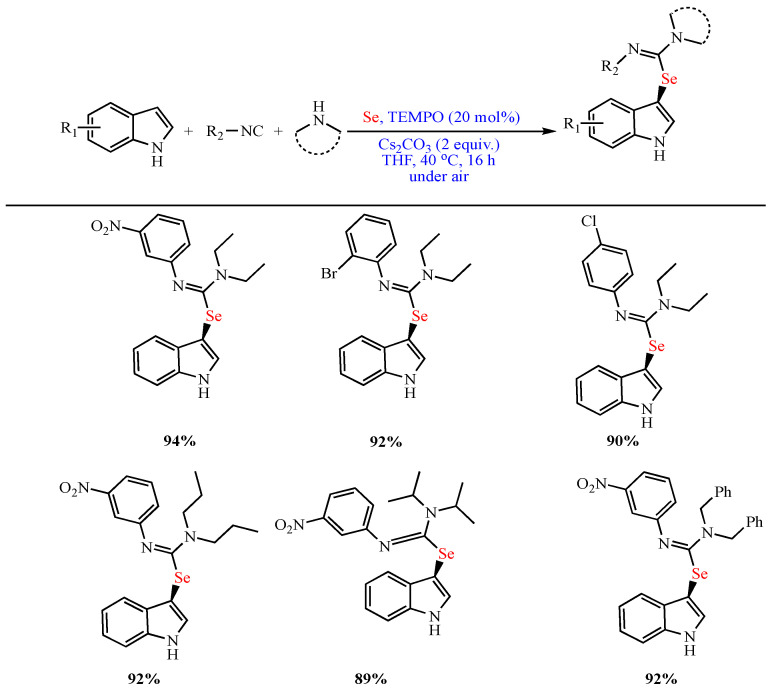
Synthesis of Se functionalization of indoles.

Liu et al. reported the multicomponent Se functionalization of indoles at the C3 under transition metal-free conditions. The reaction was carried out using elemental Se, isocyanides, amines, and indoles. The reaction proceeded under mild conditions employing O_2_ as the oxidant and TEMPO as the catalyst and encompassed a wide range of substrates in moderate to high yields (Scheme 19) [69].

**Scheme 20:**
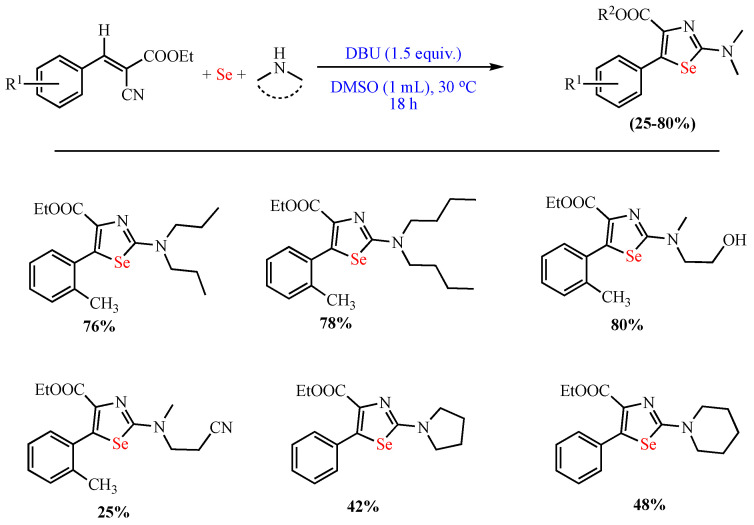
Synthesis of 2-amino-1,3-selenazoles.

In 2020, Liu et al. reported the one-pot synthesis of 2-amino-1,3-selenazoles with transition metal-free MCR of elemental Se, amines, and α,β-unsaturated isocyanides. The reaction of the elemental Se with isocyanide affords the corresponding isoselenocyanate, which undergoes intramolecular Michael cycloaddition followed by aromatization to provide 2-amino-1,3-selenazole in good yields (up to 80%) (Scheme 20) [70].

**Scheme 21:**
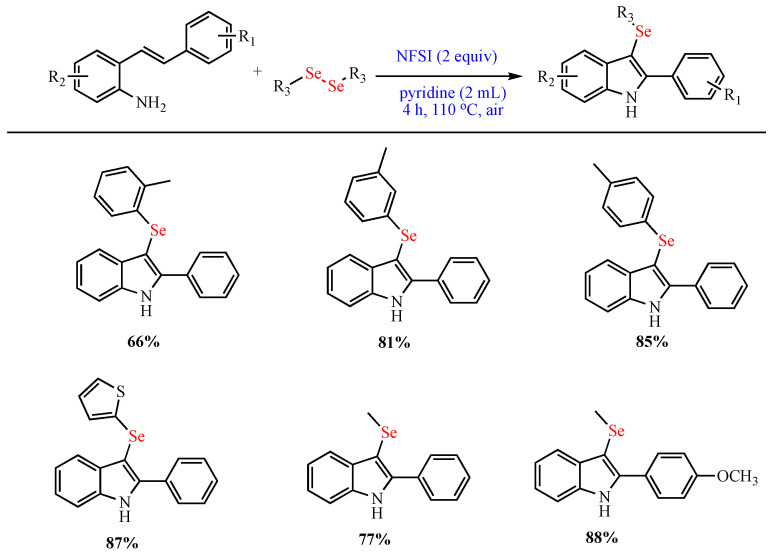
Synthesis of 3-selenylindoles.

In 2020, Zhao et al. reported the organocatalytic (*N*-fluorobenzenesulfonimide) one-pot synthesis of 3-selenylindoles through intramolecular cyclization/selenylation of 2-vinylaniline in moderate to good yield (up to 88%). The reaction was smoothly furnished, employing wide substrates and good functional group transformations, and could also be tolerated to gram scale (Scheme 21) [71].

**Scheme 22:**
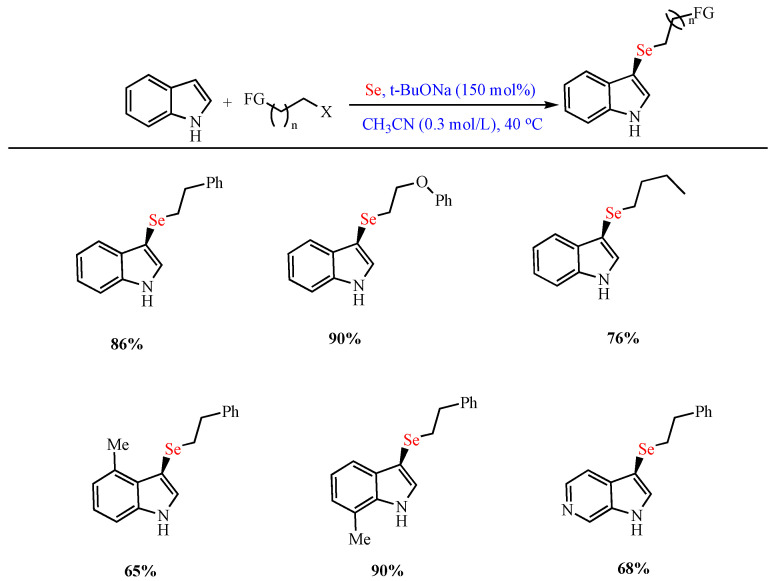
Synthesis of 3-alkylselenindole derivatives.

In 2022, Liu et al. reported the metal-free MCR synthesis of 3-alkylselenindole derivatives from elemental Se, indoles, and unactivated alkyl halides under mild conditions using *t*-BuONa as the base in CH_3_CN as the solvent at 40 °C. The reaction encompassed wide functional group tolerance and can also be applied for a large scale (>10 g) in excellent yield (>90% yield) (Scheme 22) [72].

**Scheme 23:**
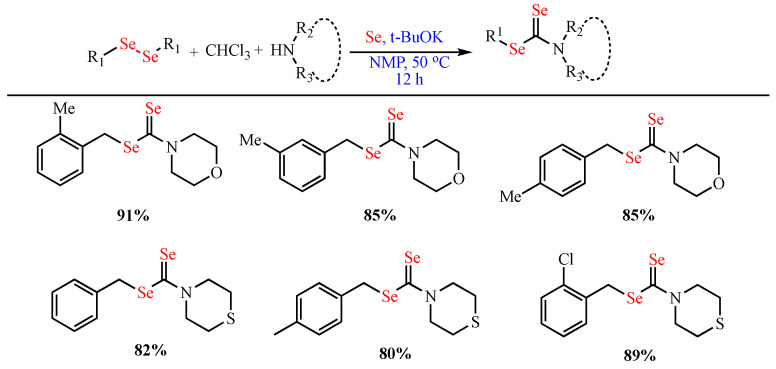
Synthesis of diselenocarbamates.

In 2021, Li et al. reported the synthesis of diselenocarbamates in good yields (up to 91%) via the one-pot MCR of elemental Se, amines, diselanes, and CHCl_3_ using *t*-BuOK as the base and NMP as the solvent at 50 °C for 12 h (Scheme 23) [73].

**Scheme 24:**
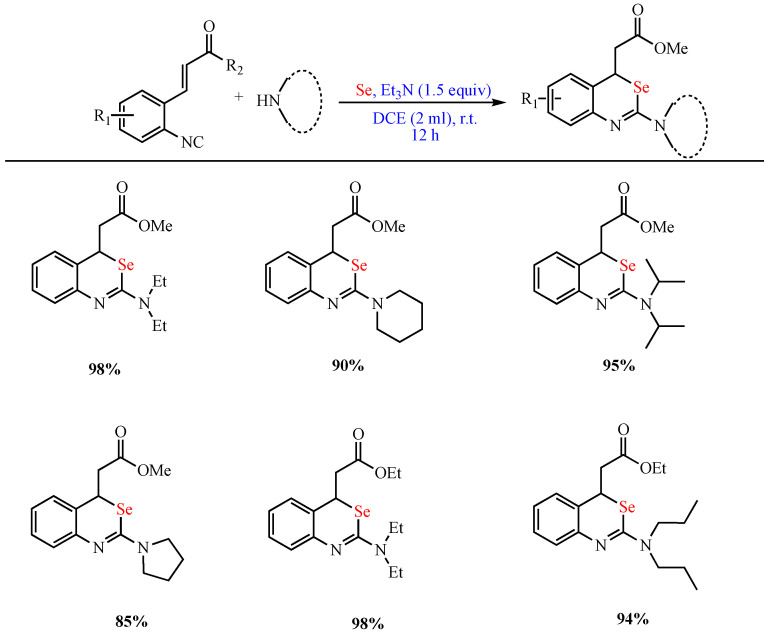
Synthesis of 2-aminobenzo[d][1,3]selenazines.

Fang et al. developed the base promoted and metal-free cascade synthesis of 2-aminobenzo[d][1,3]selenazines via reaction of elemental Se, ortho-functionalized aryl isocyanides, and amines. The reaction proceeded under basic conditions via the in situ formation of isoselenocyanates from elemental Se and subsequent intramolecular Michael addition reactions (Scheme 24) [74].

**Scheme 25:**
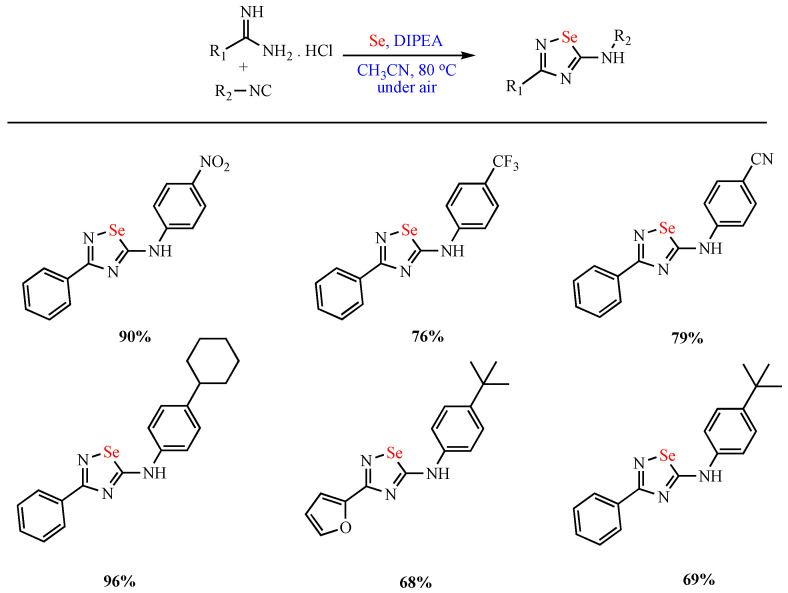
Preparation of 1,2,4-selenadiazol-5-amine derivatives.

Additionally, Fang et al. reported the metal-free preparation of 1,2,4-selenadiazol-5-amine derivatives in moderate to excellent yields (up to 96%) through the aerobic radical-cascade reactions of Se powder, isocyanides, and imidamides using O_2_ as the green oxidant. It is worth noting that the reaction H_2_O was the only byproduct obtained and the reaction encompassed good functional group tolerance and broad substrate scope. In addition, this protocol was applied for the late-stage functionalization of biologically active candidates (Scheme 25) [75].

**Scheme 26:**
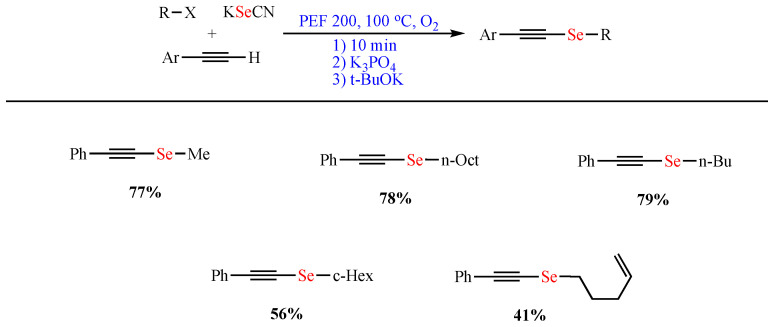
Synthesized alkynyl selenides.

Heredia et al. synthesized alkynyl selenides in moderate to good yields under aerobic metal-free conditions from the reaction of KSeCN, alkyl halides, and terminal alkynes using PEG 200 as the solvent. In this reaction, dialkyl diselenides were formed in situ from the K_3_PO_4_-assisted reaction of alkyl halides and KSeCN with terminal alkynes in the presence of *t*-BuOK (Scheme 26) [76].

**Scheme 27:**
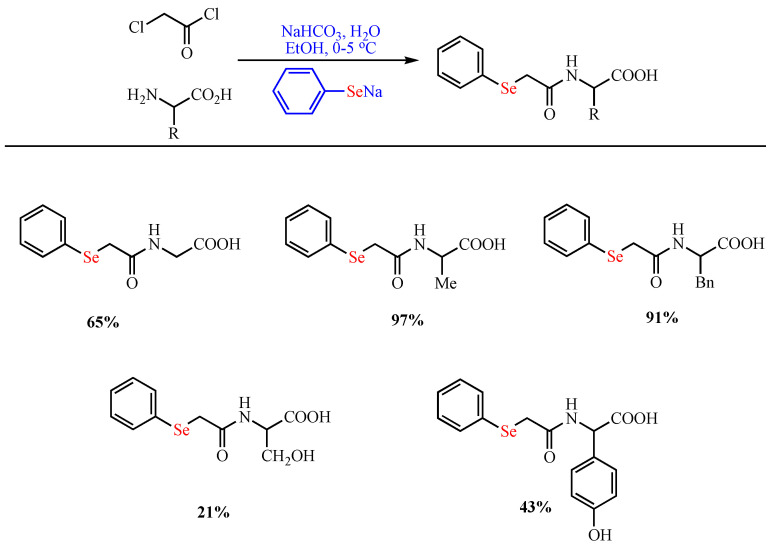
Synthesis of phenylseleno N-acetyl amino acids.

In 2013, Prabhu et al. reported the metal-free one-pot synthesis of phenylseleno N-acetyl amino acids from amino acids, chloroacetyl, and NaSePh in H_2_O: EtOH (2:1) mixed solvent (Scheme 27) [77].

**Scheme 28:**
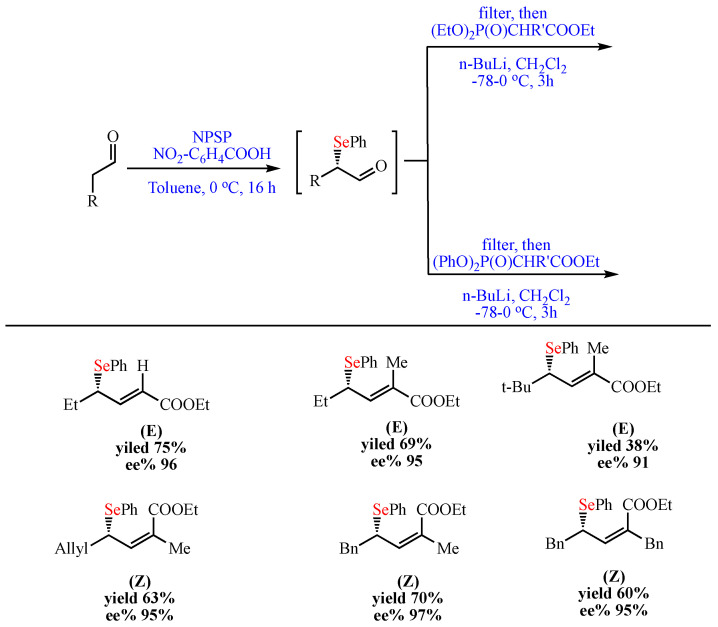
Synthesis of trisubstituted allylic selenides.

Armstrong et al. reported the synthesis of trisubstituted allylic selenides via an asymmetric, organocatalytic α-selenenylation of aldehydes using *N*-(phenylseleno)phthalimide followed by Horner–Wadsworth–Emmons olefination using and phosphonate anions (Scheme 28) [78].

**Scheme 29:**
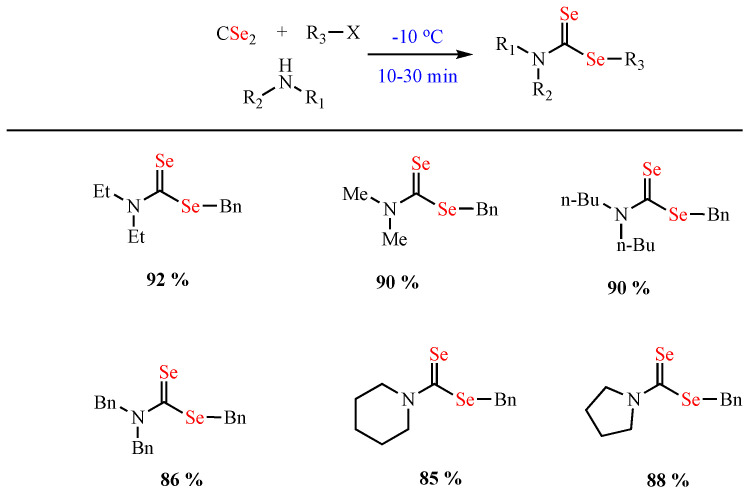
Synthesis of diselenocarbamates.

Pan et al. reported the one-pot, metal-free, and solvent-free synthesis of diselenocarbamates from the reaction of CSe_2_, alkyl halides, and amines at −10 °C (Scheme 29) [79].

**Scheme 30:**
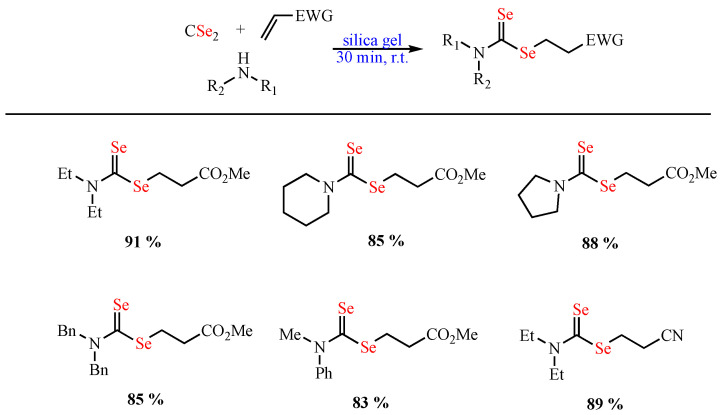
Synthesis of diselenocarbamates.

Furthermore, diselenocarbamates were synthesized from CSe2 with aliphatic amines and alkenes as electron-deficient substrates via Michael-type addition using silica gel as the media (Scheme 30) [79].

**Scheme 31:**
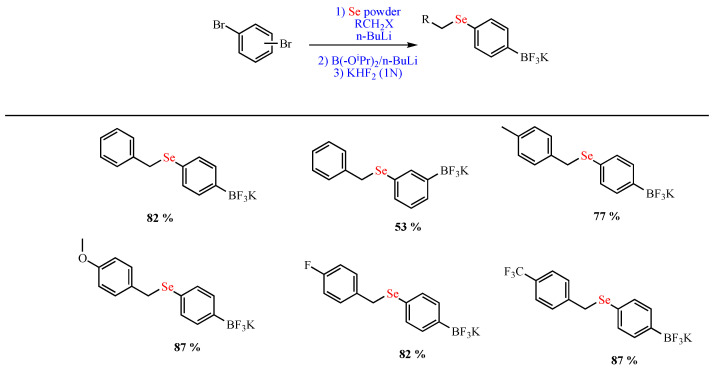
Preparation of organoselanyltrifluoroborates.

Ahn et al. reported the one-pot preparation of organoselanyltrifluoroborates from Se powder, dihalobenzenes, and alkyl halides in 56–92% yields using *n*-BuLi and boron isopropoxide at −78 °C (Scheme 31) [80].

**Scheme 32:**
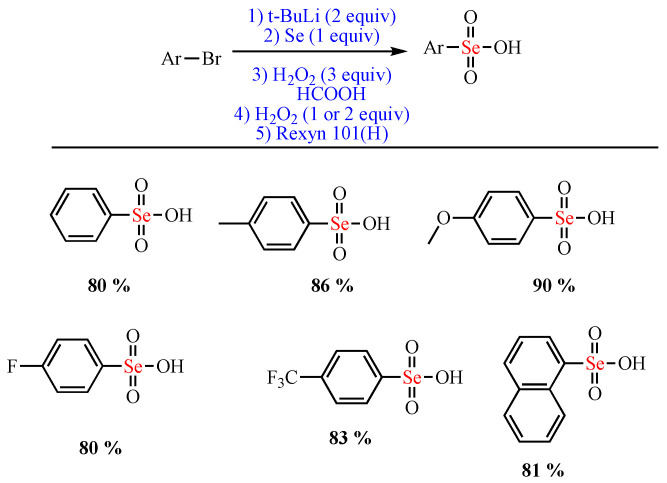
Synthesis of structurally diverse selenonic acids.

In 2021, Sands et al. reported the one-pot synthesis of structurally diverse selenonic acids in good yields (up to 90%) from elemental Se and aryl bromides. The reaction involves metalation using *t*-Butyllithium, selenation, and oxidation using H_2_O_2,_ followed by ion exchange using Rexyn 101(*H*) ion-exchange resin (Scheme 32) [81].

**Scheme 33:**
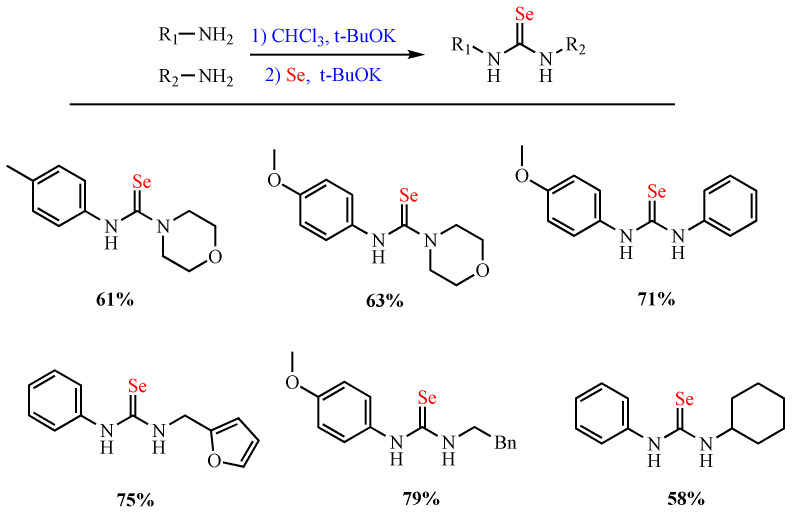
Synthesis of unsymmetrical selenoureas and cycloselenoureas.

In 2020, Wu et al. disclosed the one-pot multicomponent synthesis of unsymmetrical selenoureas and cycloselenoureas from selenium powder, CHCl_3_, and two different amines using *t*-BuOH as the base at 50 °C for 3 h in moderate–good yields (up to 86%) (Scheme 33) [82].

**Scheme 34:**
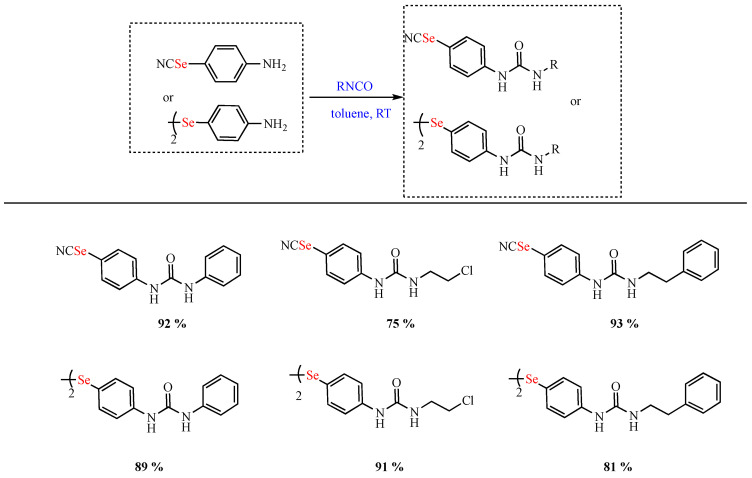
Synthesis of urea-based selenocyanates and symmetrical diselenides.

In 2022, Shaaban and colleagues reported the development of urea-based selenocyanates and symmetrical diselenides in good yields (up to 93%) using 4-selenocyanatoaniline and 4,4′-diselanediyldianiline, respectively, and commercially available isocyanates in toluene (Scheme 34) [83].

**Scheme 35:**
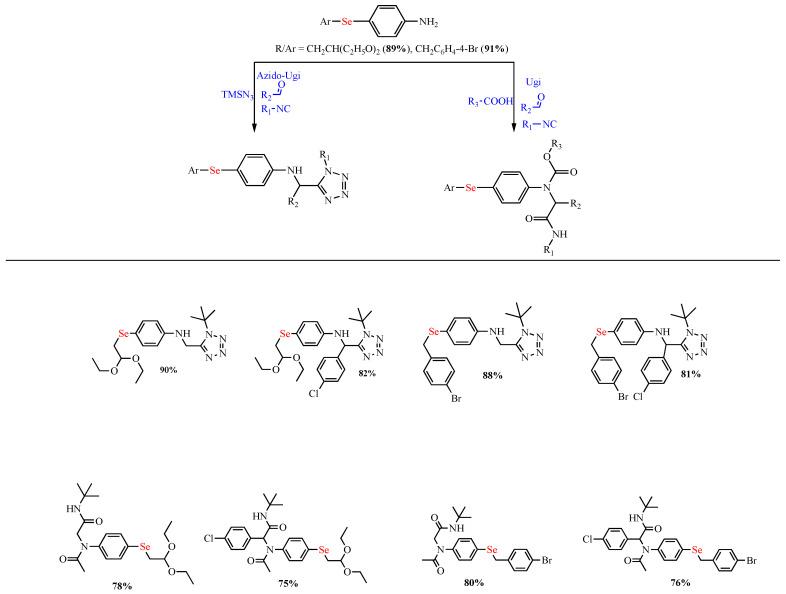
Synthesis of peptide-like and tetrazole-based redox-active multifunctional OSe compounds.

In 2021, Shaaban et al. and his group developed of peptide-like and tetrazole-based redox-active multifunctional OSe compounds via multicomponent Ugi and azido-Ugi reactions. The reaction included novel Se-based aniline building blocks to incorporate the Se redox center into the backbone of the Ugi/Ugi-azide structurally diverse product’s tail. Indeed, the reactions were carried out under mild conditions using DCM and MeOH as the solvent for the Ugi and Azido-Ugi reactions, respectively (Scheme 35) [12].

**Scheme 36:**
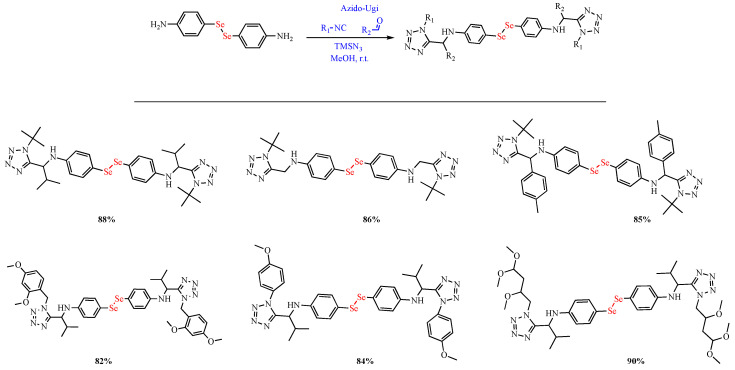
Synthesis of tetrazole-based symmetrical diselenides and selenoquinones compounds.

Furthermore, Shaaban et al. reported the combinatorial one-pot synthesis of tetrazole-based symmetrical diselenides and selenoquinones compounds via azido-Ugi and sequential nucleophilic substitution methodology (Scheme 36) [84].

**Scheme 37:**
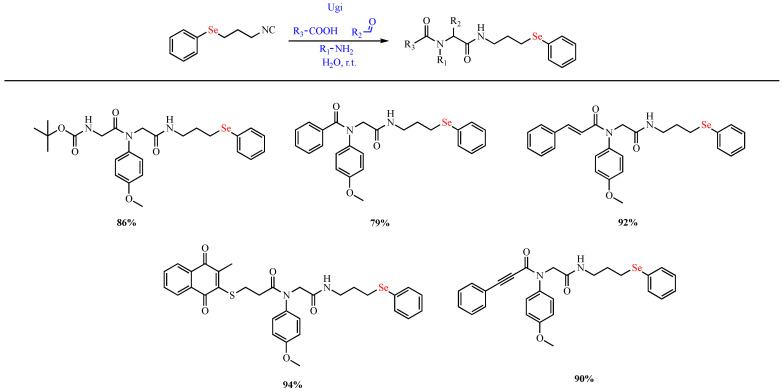
Synthesis of different Se peptidomimetic compounds.

Moreover, Shaaban et al. also reported the synthesis of different Se peptidomimetic compounds employing the Ugi isocyanide-based MCR using the Se-based isonitrile 3-isocyanopropyl(phenyl)selane. The reaction was achieved under mild conditions using H_2_O as the solvent in good yields (up to 94%) (Scheme 37) [85].

**Scheme 38:**
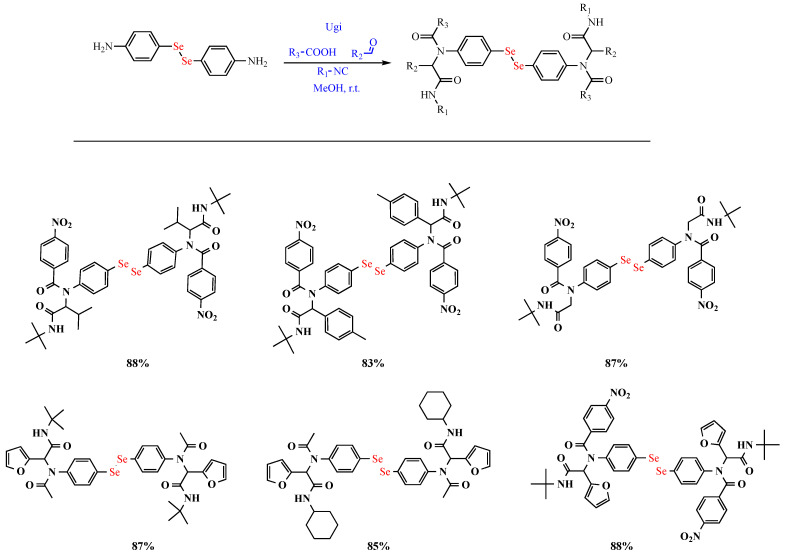
Synthesis of symmetrical diselenide.

Additionally, Shaaban et al. reported the synthesis of symmetrical diselenide via one-pot Ugi using 4-(2-(4-aminophenyl)diselanyl)benzenamine as key synthon, which in turn allowed the access to the diselenide scaffolds (Scheme 38) [86].

**Scheme 39:**
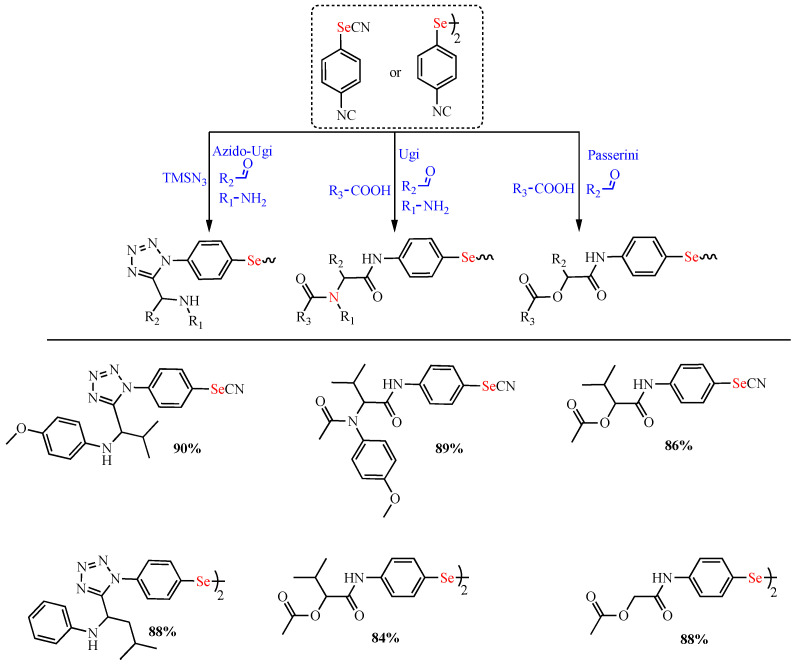
The synthesis of new selenocyanate isocyanide and diselenide diisonitrile and explored their reactivities in Passerini, Ugi, and Azido-Ugi reactions.

In 2021, Shaaban et al. reported the synthesis of new selenocyanate isocyanide and diselenide diisonitrile and explored their reactivities in Passerini, Ugi, and Azido-Ugi reactions. OSe-based pseudopeptides, peptidomimetics, and tetrazoles were obtained in good yields (up to 94%) (Scheme 39) [87].

**Scheme 40:**
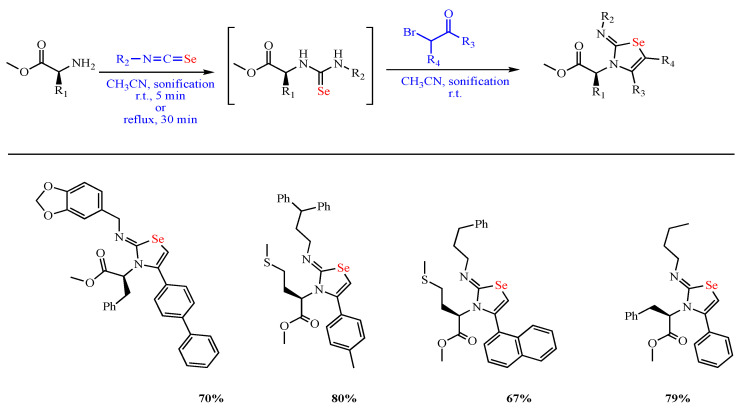
Synthesis of chiral 2-iminoselenazolines.

Chang et al. reported the three components regioselective one-pot synthesis of chiral 2-iminoselenazolines by sonication from L-aminoester, isoselenocyanate, and α-bromoketone. In this reaction, selenazoles are obtained by Hantzsch reaction of selenoureas, generated in situ from the reaction of isoselenocyanate and L-amino esters, with α-bromoketones proceeded smoothly under ecofriendly conditions, i.e., at room temperature and under ultrasonication (Scheme 40) [88].

**Scheme 41:**
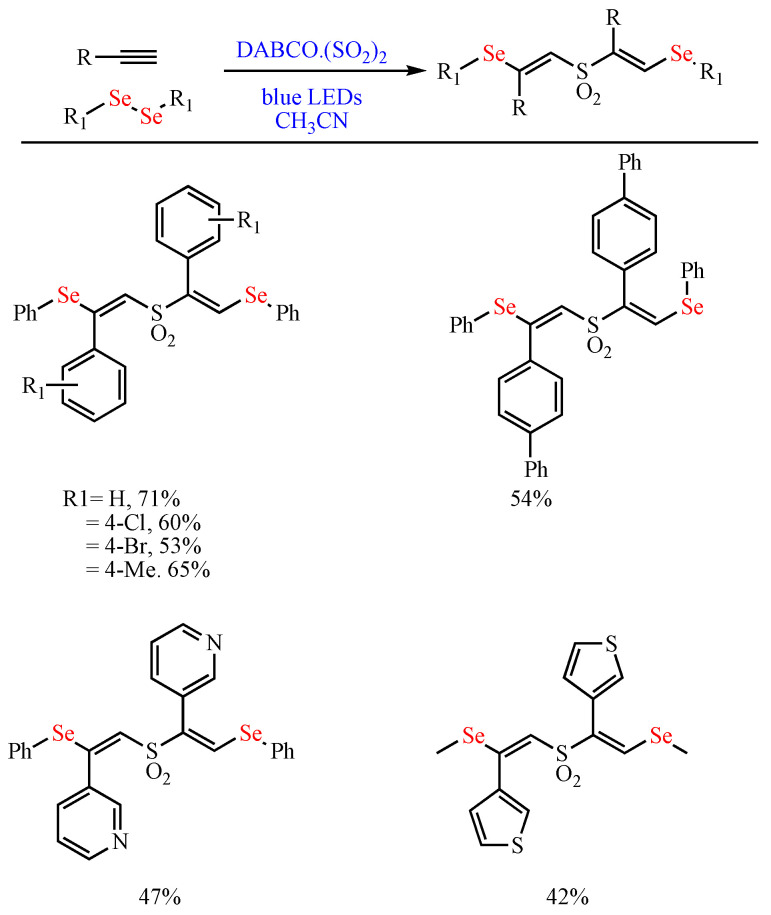
Synthesis of β-sulfonylvinylselanes.

Chen et al. reported the construction of β-sulfonylvinylselane bond via the visible-light mediated MCR cascade of diselenides, alkynes, and SO_2_. In this reaction, novel class of β-sulfonylvinylselanes in high selectivity for E configuration and in moderate yields (up to 71%) (Scheme 41) [89].

### 2.3. Multicomponent Polymerization Synthesis of OSe Compounds

**Scheme 42:**
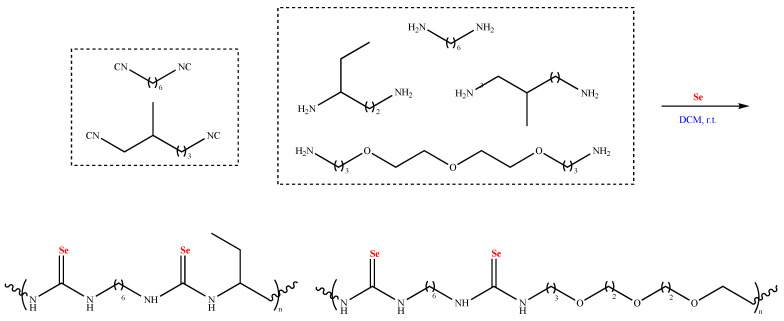
The multicomponent polymerization synthesis of polyselenoureas.

In 2018, Tuten et al. reported the multicomponent polymerization reaction of elemental Se, amines, and isocyanides to form polyselenoureas in one step and at room temperature using DCM as the solvent (Scheme 42) [90].

**Scheme 43:**
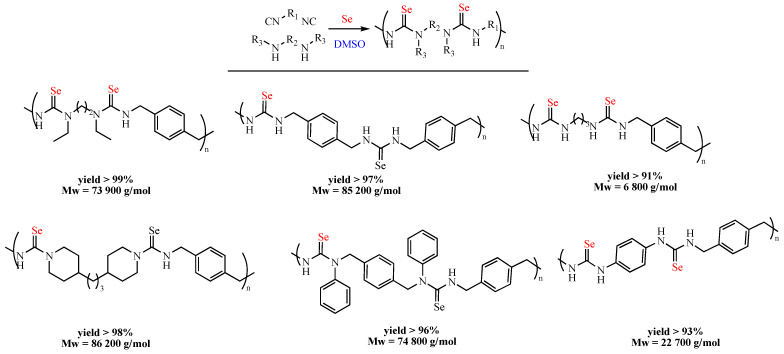
Synthesis of functionalized and structurally diverse polyselenoureas.

In 2019, Wu et al. reported the synthesis of functionalized and structurally diverse polyselenoureas via catalyst-free and solvent-free MC polymerizations of elemental Se, diisocyanides, and aliphatic/aromatic diamines. It is worth noting that the obtained polyselenoureas encompassed enhanced thermal stability and solubility, long-term stability, and the potential extraction of gold ions (Au^3+^) from mixed-metal ion solutions (Scheme 43) [91].

**Scheme 44:**
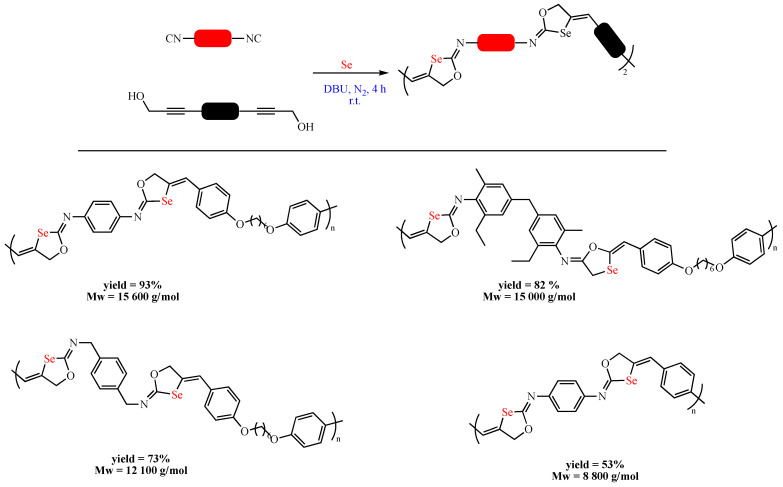
Synthesis of alicyclic poly(oxaselenolane)s.

In 2021, the same group by Tang et al. reported the synthesis of alicyclic poly(oxaselenolane)s at room temperature via metal-free multicomponent polymerizations of elemental Se dipropargyl alcohols and diisocyanides. It is worth noting that poly(oxaselenolane)s were obtained in high yields (up to 93%), high Se contents (up to 33.7 wt %), high molecular weights (up to 15 600 g/mol), and high thermal and chemical stability as well as excellent light refractivity, good solubility, and processability. Furthermore, the polymerization reaction encompassed a broad scope of the diisocyanides (e.g., benzyl and aromatic) as well as various dipropargyl alcohols (Scheme 44) [92].

## 3. Conclusions

In conclusion, the direct one-pot multicomponent synthesis of OSe compounds has emerged as a potential and atom-economic strategy. Furthermore, different metal-free, as well as metal-catalyzed, reactions evolved during the last decade. These approaches open new scopes for synthesizing OSe compounds, a group of compounds with attractive chemical, biological, and physical activities. Without a doubt, novel and improved strategies for synthesizing OSe compounds will also be released soon, addressing challenges such as site-selectivity, late-stage selenylation of natural products, and complex molecules.

## Data Availability

The data presented in this study are available on request from the corresponding author.
